# Novel drugs targeting genetic variants: current applications and future prospects in heart failure treatment

**DOI:** 10.3389/fmolb.2026.1781854

**Published:** 2026-03-05

**Authors:** Xiang Li, Suwen Bai, Yumei Luo

**Affiliations:** 1 Guangdong Medical University, Zhanjiang, China; 2 Central Laboratory, The Second Affiliated Hospital, School of Medicine, The Chinese University of Hong Kong, Shenzhen & Longgang District People’s Hospital of Shenzhen, Shenzhen, China; 3 The Second Affiliated Hospital, School of Medicine, The Chinese University of Hong Kong, Shenzhen & Longgang District People’s Hospital of Shenzhen, Shenzhen, China

**Keywords:** application, chronic heart failure, novel drugs, targeted genetic mutation drugs, treatment

## Abstract

Chronic heart failure (HF) is a common and frequently occurring disease worldwide, and its traditional treatment methods are undergoing earth-shaking changes. This study analyzes novel drugs targeting genetic mutations that have been launched in recent years, such as sodium-glucose co-transporter 2 inhibitors, angiotensin receptor neprilysin inhibitors, soluble guanylate cyclase, cardiac myosin activators, potassium channel openers, and vasopressin receptor antagonists, compares the drug action mechanisms, key clinical trial data in the research, and the application of real-world research, and discusses the clinical value and existing problems of new drugs.

## 1 Introduction

Heart failure (HF) is a major global public health challenge, with the incidence increasing by 2% each year. By 2030, nearly 80 million people will have HF (Tamargo et al., 2019). Although traditional treatments (ACEI/ARB, β-blockers, aldosterone antagonists) have significantly improved patient prognosis, the 5-year mortality rate of heart failure patients is still as high as 30%–50%, and the patient re-hospitalization rate is also high. The above situation shows the limitations of existing treatment strategies, and mechanism innovation is needed to break through the treatment bottleneck. In recent years, the number of new drug developments has increased exponentially, and the targets have been shifting from neuroendocrine regulation to multi-pathway and multi-link mechanisms. In recent years, the number of new drug developments has increased exponentially, and the targets have been shifting from neuroendocrine regulation to multi-pathway and multi-link pathological mechanisms, such as epigenetics and cardiovascular medicine. Genome-wide association studies (GWAS) have identified numerous loci associated with HF risk, cardiomyocyte contractility, and fibrosis, implicating pathways involved in cardiac development, ion handling, and stress signaling (Aragam et al., 2022). Genetic polymorphisms in drug target genes. For example,Sodium-glucose cotransporter 2 inhibitors (SGLT2i) can affect myocardial active metabolism through Adenosine 5‘-monophosphate activated protein kinase‌(AMPK)and Recombinant Sodium/Hydrogen Exchanger 1(NHE1) (Zhang et al., 2023). Angiotensin receptor neprilysin (ARNI)can circulate the levels of neprilysin (NEP). This elevated NEP pool competes for binding with NPR3,inducing cardiomyocyte contractility. The emergence of new drugs such as myo-centric amti-vimetcarbim and potassium channel opener (Kchan-nelopetnoseu-taior) is ushering in a new era of targeted genetic mutation drugs in the field of heart failure treatment (Ge et al., 2025). Atrial natriuretic peptide clearance receptor (NPR3) could affect drug efficacy and safety profiles. Therefore, these genetic and epigenetic underpinnings might be critical for guiding the development of novel, targeted genetical therapies of heart failure (Bekfani and Schulze, 2024). This review analyzes these novel drug therapeutics and their association with genetic and epigenetic mechanisms, through the evidence from pivotal clinical trials, to discuss their current application, unresolved challenges, and the predicting future development trends, in order to pave the way for targeted genetic mutation drugs medicine in heart failure.

## 2 Novel drug targeting genetic variants types and mechanisms of action

### 2.1 Sodium-glucose cotransporter 2 inhibitors (SGLT2i)

#### 2.1.1 Detailed explanation of the mechanism of action of SGLT2i

The benefits of SGLT2i in heart failure are independent of the effects of blood glucose (Manu et al., 2021). Osmanska and Jhund (2019) pointed out that the traditional mechanism is that it can inhibit proximal renal tubular SGLT2, resulting in reduced glucose reabsorption, excreting 70–100 g of urine glucose per day, producing an osmotic diuretic effect induced by active diuretics, thereby reducing cardiac preload (Osmanska and Jhund, 2019). In recent years, studies on its non-metabolism-dependent myocardial protection mechanism are as follows: Huet et al. (2023) pointed out that reducing sodium overload in myocardial cells and upregulation of NHE1 are its main mechanisms for preventing myocardial fibrosis, and maintaining effective utilization of the heart through metabolic conversion of fatty acid oxidation to glucose utilization (Huet et al., 2023). Nikolaidou et al. (2024) pointed out that inhibiting NADPH oxidase activity reduces the production of mitochondrial ROS and ultimately reduces myocardial fibrosis (Nikolaidou et al., 2024); Ma (2019) pointed out that activation of the AMPK/mTOR pathway will promote autophagy to clear exhausted mitochondria (Ma, 2019). The efficacy of SGLT2i in heart failure may still be confusing in the process of benefiting HFpEF patients, such as reducing inflammation of visceral fat and improving diastolic function (Wei, 2018). The author believes that the cardiac multiple benefit characteristics of this type of drug provide a paradigm for achieving cardiac treatment based on all evidence, and studying the cell subset-specific network of this drug will lead to more clues for future heart failure treatment based on single-cell sequencing, thereby achieving more accurate individualized heart failure treatment.

**FIGURE 1:**
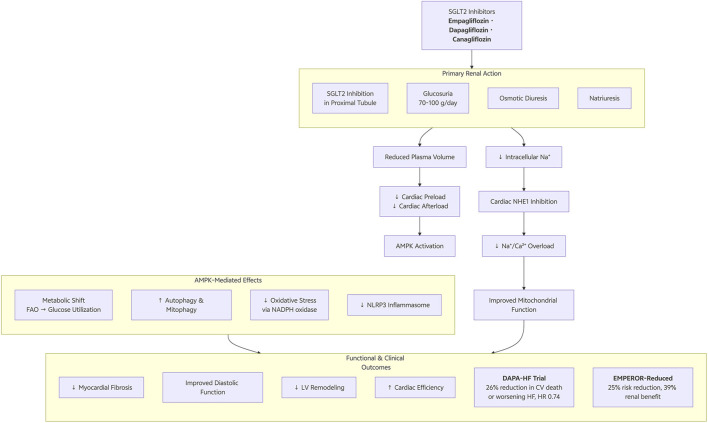
From renal natriuresis to cardiac AMPK activation and NHE1 inhibition.

SGLT2i has cardioprotective effects, which works in part by mediating the effect of AMPK and NHE1 inhibition (Figure 1). Empagliflozin stimulates AMPK in the heart tissues by increasing autophagy and mitochondrial biogenesis and decreasing the levels of oxidative stress. The change in metabolism as fatty acids become replaced by glucose enhances effciency in the myocardium SGLT2i under energy-deprived conditions (Jaishy et al., 2021). Besides, SGLT2i decreases NHE1 in cardiomyocytes, thus reducing the effects of mitochondrial apoptotic signal (Uthman et al., 2019). Empagliflozin maintains cardiac functioning through NHE1 inhibition (Baartscheer et al., 2017).

#### 2.1.2 Introduction of representative drugs

At present, the approved SGLT2i for heart failure include dapagliflozin, empagliflozin and sopagliflozin, each with different clinical positioning. Dapagliflozin was established as the first SGLT2i for heart failure by the DAPA-HF study, which reduced the risk of cardiovascular death or worsening heart failure with reduced ejection fraction (HFrEF)patients by 26% (Weisert et al., 2022). The strong renal protection function of empagliflozin shown in the EMPRIOR-Reduced study reduced the absolute risk of proteinuria progression by 39%. Sopagliflozin, as a dual SGLT1/2 inhibitor, was effective in improving the symptoms of acute heart failure in the early stage in SOLOIST-WHF, but there were also concerns about more gastrointestinal side effects (Nadur et al., 2021). The tissue selectivity of different drugs is different. The tissue selectivity of dapagliflozin over SGLT2 is 1,200 times, while that of empagliflozin is 2,500 times (Haley et al., 2021), which may have an important impact on its metabolic effects. The author believes that head-to-head clinical studies will be needed in the future to clarify the differential efficacy of different drugs. At the same time, it is necessary to explore individualized drug treatment strategies based on different patient phenotypes (such as renal function and comorbidities) in order to maximize patient efficacy.

#### 2.1.3 Display of relevant clinical research results

The evidence-based evidence for the use of SGLT2i in heart failure has reached the level of complete clinical evidence. The DAPA-HF trial confirmed the primary value of dapagliflozin for the treatment of heart failure. The trial included 4,744 HFrEF patients and confirmed that it could reduce the risk of cardiovascular death and worsening heart failure by 26% (p < 0.001) (Hu et al., 2025). The EMPEROR-Reduced trial further expanded the indications of empagliflozin. In addition to reducing the risk of the primary endpoint, it also slowed the risk of worsening renal function by 39% (Denda et al., 2024). The SOLOIST-WHF trial showed that sogliflozin’s unique advantage in patients with acute heart failure is that it can quickly improve patients’ dyspnea (45% relief within 72 h), but it is necessary to pay attention to the increased risk of ketoacidosis compared with the placebo group (0.7% vs. 0.2%) (Matsuo et al., 2020). Machaj et al. (2019) based on the study, the DELIVER trial showed for the first time that SGLT2i was beneficial for HFpEF, with dapagliflozin reducing the risk of the composite endpoint by 13% (p = 0.001), while the efficacy trend of empagliflozin for HFpEF was consistent with that for HFpEF (HR = 0.92, p = 0.18) (Machaj et al., 2019). The author believes that the above studies have influenced the formulation of guidelines for the treatment of heart failure, and have made SGLT2i a consensus drug in the main battlefield from a niche second-line drug. However, the limitations of the existing evidence include the lack of population data from Asia and long-term safety data. In the future, more real-world studies and precise subgroup analyses guided by biomarkers are still needed.

### 2.2 Angiotensin receptor neprilysin inhibitor (ARNI)

#### 2.2.1 Unique mechanism of action of ARNI

Kinoshita et al. (2024) indicated that ARNI has dual effects; this is due to its capability of blocking angiotensin II receptor (AT1R) and neprilysin (NEP) in the regulation of the RAAS system and the natriuretic peptide pathway. Its active metabolite, LBQ657, has an affinity for AT1R 3 times greater than that of valsartan, making it competent to elevate the level of endogenous natriuretic peptides by 2–3 times (Liu et al., 2024). These mechanisms were established in the PARADIGM-HF trial to achieve a 20 percent reduction in the risk of cardiovascular death in HFrEF patients (p < 0.001) and are more effective in enhancing remodeling of the left ventricle compared to enalapril (left ventricular ejection fraction increased by 3.4% and 1.0%, respectively) (Chen et al., 2018). It is worth mentioning that Lu et al. (2018) remarked that the dual blood pressure regulation of ARNI can lessen the usage of diuretics, yet it must be cautioned that it can only be administered to patients with hypotension to a limited extent (Kruchinkina et al., 2018). The author thinks that the multi-target intervention model of ARNI offers a novel concept for the treatment of heart failure; however, the therapeutic administration could result in long-term compensatory upregulation of NEP, hence there is a need to pursue short bursts of drug administration to further enhance the therapeutic effects and drug resistance ratio.

**FIGURE 2:**
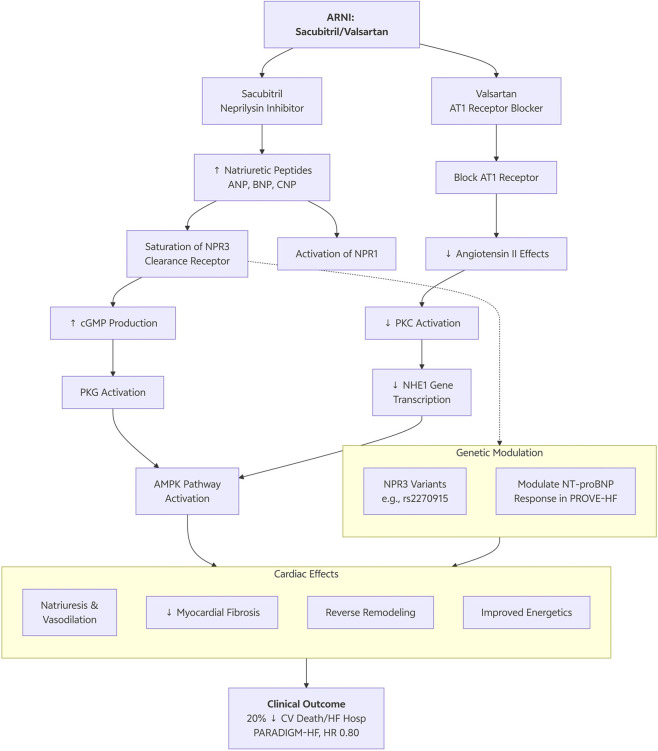
Dual modulation of the NPR3 clearance pathway and NHE1 gene transcription in heart failure.

ARNI regulates neurohormonal axes and has an effect on myocardial gene expression (Figure 2). The inhibitory effect of ARNI on angiotensin II inhibits protein kinase C (PKC) activation, which is an established activator of NHE1 expression. Valsartan component is a direct antagonist of AT1R, decreasing the transcription and activity of NHE1, and having antiremodeling effects (Zhao et al., 2020).

NPR3 is a key controller of the bioavailability of natriuretic peptide (NP) thereby taking up NPs (ANP, BNP, CNP) and degrading them, which suppresses the signaling. NPR3 upregulation helps cause NP resistance in clearance, thus accelerating the beneficial effects of endogenous NPs such as vasodilatory, natriuretic, and anti-fibrotic effects (Lu et al., 2018). ARNI is a neprilysin NEP inhibitor. NEP is a membrane-bound endopeptidase that degrades active NPs. By inhibiting NEP, sacubitril increases circulating levels of NPs (Potter, 2011). NPs pool competes for binding with NPR3, effectively saturating the clearance pathway and enhancing signaling through the beneficial NPR1 receptor.

#### 2.2.2 Analysis of clinical advantages

Due to the dual mechanism of action of ARNI, PARADIGM-HF showed that it could significantly reduce the risk of cardiovascular death by 20% (hazard ratio = 0.80, p < 0.001) and left ventricular ejection fraction by 3.4% in HFrEF patients compared with the enalapril group, which was 1.0% higher (Kusumoto et al., 2018). Kruchinkina et al. (2018) pointed out that the PIONEER-HF trial in acute heart failure showed that its early use in acute heart failure could rapidly reduce NT-proBNP by 42% and shorten the length of hospital stay by 2.3 days (Kruchinkina et al., 2018). Zhao et al. (2020) showed based on real-world results that it can significantly reduce the all-cause mortality rate of heart failure patients by 18% and the re-hospitalization rate by 25% compared with enalapril (Zhang et al., 2021). Chen et al. (2018) pointed out that the phenomenon of insufficient dosage in Asian populations (the average dose of ARNI only reached 68% of the guideline recommendation) may compromise its efficacy (Chen et al., 2018). The author believes that it has significant advantages in reducing mortality, improving symptoms and delaying disease progression. However, the risks of hypotension (12.4% vs. 8.6% for enalapril) and hyperkalemia (5.5% vs. 4.3%) need to be optimized through individualized dosage and blood potassium monitoring. In the future, the synergistic effect of ARNI combined with SGLT2i can be explored to expand the beneficiary population.

#### 2.2.3 Presentation of clinical application data

From the clinical application of ARNI, we can see that it can greatly improve the prognosis of patients. In the PARADIGM-HF trial, sacubitril-valsartan reduced the risk of CV death in heart failure with reduced HFrEF patients by 20% (HR = 0.80, p < 0.001) and the all-cause mortality rate by 16% (Zhao et al., 2020). Momin and Lotankar (2018) pointed out that based on real-world medication data, its application can reduce the rate of re-hospitalization for heart failure by 25%, the number of emergency visits by 32%, and the annual increase in global prescriptions by 35%, but the trend of insufficient dosage in the Asian population is obvious (Momin and Lotankar, 2018). Yang et al. (2023) confirmed that early application in acute heart failure can quickly reduce NT-proBNP levels by 42% and shorten hospitalization time by 2.3 days. The incidence of gastrointestinal adverse reactions (15.8%) in the study was not significantly different from that of enalapril (12.4%), and the risk of hypotension should be noted (12.4% vs. 8.6%) (Yang et al., 2023). The author believes that the difference between the benefits of ARNI in the real world and in clinical trials may be related to insufficient dose adjustment and differences in patient selection. In the future, intelligent algorithms should be used to guide dose titration to optimize clinical practice.

### 2.3 Soluble guanylate cyclase stimulators (vericiguat)

#### 2.3.1 Explanation of the working principle

Vericiguat also activates sGC directly to elevate cGMP, enhancing the activities of the NO-cGMP pathway, and the small hint of it is that it does not have to depend on endogenous NO (Paolillo et al., 2024). VICTORIA demonstrated that the risk of the occurrence of major endpoint events in chronic heart failure can be decreased by 10 per cent (HR = 0.90, p = 0.009), though it can slow down the decline in renal functions by 23 per cent (Jian et al., 2019). According to Karoli et al. (2021), due to the pharmacokinetic properties, it can be administered once daily as an oral drug; it can be used with an oral beta-blocker to be synergistic (VITALITY-HF6, HR = 0.78, p = 0.015) (Karoli et al., 2021). We are anticipating the application of the technology of genetic testing in the future so as to get accurate medication and to explore more possibilities of making a union with ARNI.

Vericiguat seasonally activates the NO-sGC-cGMP reactant network, downstream of which it crosses the AMPK signaling cascade, increasing the concentration of cGMP-dependent protein kinase (PKG). PKG activates and phosphorylates AMPK to produce a cardioprotective signal that increases endothelial activity and decreases cardiac hypertrophy (Follmann et al., 2021). This cGMP-AMPK pathway enhances energy homeostasis and alleviates oxidative stress. In addition, vericiguat reduces the effects of NP clearance caused by high NPR3. Vericiguat offers an alternative pathway of cGMP increase in a state of NP deficiency (Armstrong et al., 2020). Vericiguat Soluble Guanylate Cyclase Parallel Pathway Activator stimulates cGMP bypassing the NP-receptor system, but possible functional interaction with NPR3 activity state is not studied (Ezekowitz et al., 2022).

#### 2.3.2 Clinical research evidence support

In the VICTORIA trial (Solomon et al., 2022), the clinical benefits of vericiguat in the treatment of chronic heart failure were confirmed by evidence, including 5,050 patients with chronic heart failure. Vericiguat can reduce the risk of cardiovascular death or heart failure hospitalization by 10% (HR = 0.90, p = 0.009) and slow the deterioration of renal function by 23%. Its efficacy is also significant in patients with HFrEF and HFpEF (HR = 0.88, 0.92) (Li, 2018). In the VITALITY-HF trial (Metra et al., 2021), combined therapy with beta-blockers reduced NT-proBNP levels by 41% and improved exercise tolerance (6-min walking distance increased by 28 m). However, its use in patients with low blood pressure (<100 mmHg) should be used with caution, as the incidence of hypotension in the trial was 11.2%, which was no significant difference from the placebo control group (Metra et al., 2022). The author believes that vericiguat has added a new pharmacological option for the treatment of heart failure, but its long-term safety (such as melanoma risk signals) needs to be confirmed by extended follow-up. The additive effect of vericiguat combined with SGLT2i and its precise application strategy guided by biomarkers can be further explored.

### 2.4 Other new drugs (levosimendan, ivabradine, etc.)

#### 2.4.1 Levosimendan

Levosimendan can simultaneously improve myocardial blood perfusion by enhancing the calcium sensitivity of cardiac troponin C and activating ATP-sensitive potassium channels (KATP) to produce a positive inotropic effect without increasing myocardial oxygen consumption (Yang et al., 2019). Katsiki et al. (2024) pointed out based on the SURVIVE trial that it can reduce the mortality of patients with acute heart failure by 26% (p = 0.028) for 180 days, and REVIVEII confirmed that it can rapidly improve the hemodynamics of patients with heart failure (increase cardiac output by 28%) (Katsiki et al., 2024). Li (2018) pointed out that real-world evidence of levosimendan also suggests that its application in patients with low cardiac output syndrome can reduce the need for mechanical circulatory support (Li, 2018). However, its disadvantage is that it has a long half-life (75–80 h), and sustained effectiveness can be achieved with a single intravenous infusion, but it increases the risk of ventricular arrhythmias (incidence 5.6% vs. 3.8% in the dobutamine group). The author believes that the unique advantages of levosimendan in acute heart failure will make it a clinically advantageous choice, but long-term safety data are lacking. In the future, personalized medication patterns with biomarkers will be explored, and the effects of combination with new positive inotropic drugs such as omecamtivmecarbil will be explored.

#### 2.4.2 Ivabradine

Ivabradine slows the heart rate by specifically inhibiting the If channel in the sinoatrial node, independent of beta-blockers (Li et al., 2024). Yang (2019) show that the usage rate in patients with heart failure who are intolerant to beta-blockers is as high as 23%, but only 58% used the appropriate dose. There is no obvious benefit in patients with a heart rate <70 bpm (SHIFT subgroup analysis HR = 1.05), suggesting that the application of ivabradine requires precise targeting of the target group (Magnesa et al., 2022). The author believes that ivabradine, as the only heart rate regulator currently available, has expanded the perspective on the treatment of heart failure. In the future, it is necessary to find and verify the possibility of optimizing the dose of ivabradine based on heart rate variability. It is also necessary to find and evaluate its optimal use on heart rate in combination with SGLT2i.

## 3 Current status of clinical application of new drugs

### 3.1 Progress in clinical research

#### 3.1.1 Summary of key clinical studies of new drugs

The clinical research of new drugs has formed a complete evidence system. The following is a summary of the core trial data (Table 1).

**TABLE 1:** Summary of key clinical studies of new drugs.

Study name	​Medications/ Interventions	Indications	Primary end point	Results	​HR (95% CI)	p-value	Year
DAPA-HF	Dapagliflozin	HFrEF	Risk of cardiovascular death or worsening heart failure	26% reduction	0.74 (0.65–0.85)	<0.001	2019
EMPEROR-reduced	Empagliflozin	HFrEF	Risk of cardiovascular death or worsening heart failure	Reduced by 25%; delayed the risk of renal function deterioration by 39%	0.75 (0.65–0.86)	<0.001	2022
SOLOIST-WHF	Sogliflozin (SGLT1/2i)	Acute heart failure	Relief of dyspnea within 72 h	45% remission rate vs. 28% in the placebo group	—	—	2020
DELIVER	Dapagliflozin	HFpEF	Composite endpoint (cardiovascular death/worsening of heart failure)	13% reduction	0.87 (0.77–0.98)	0.001	2023
PARADIGM-HF	Sacubitril/valsartan (ARNI)	HFrEF	Risk of cardiovascular death or heart failure hospitalization	20% decrease; left ventricular ejection fraction increased by 3.4% vs. 1.0% with enalapril	0.80 (0.73–0.87)	<0.001	2014
PIONEER-HF	Sacubitril/valsartan (ARNI)	Acute heart failure	Decreased NT-proBNP levels	42% reduction	—	—	2017
VICTORIA	Vericiguat	HFrEF/HFpEF	Risk of cardiovascular death or heart failure hospitalization	10% reduction	0.90 (0.82–0.99)	0.009	2021
VITALITY-HF	Vericiguat + beta-blocker	HFrEF	NT-proBNP decreases	Combination therapy reduced the risk by 41%	—	—	2021
SURVIVE	Levosimendan	Acute heart failure	180-day mortality rate	26% reduction	0.74 (0.58–0.95)	0.028	2002
REVIVE-II	Levosimendan	Acute heart failure	Improved hemodynamics (increased cardiac output)	28% increase	—	—	2002
SHIFT	Ivabradine	HFrEF (heart rate ≥70 bpm)	Risk of cardiovascular death or heart failure hospitalization	18% reduction	0.82 (0.75–0.90)	<0.0001	2010

SGLT2i has been confirmed as a cornerstone therapy by DAPA-HF (McMurray et al., 2019) and EMPEROR-Reduced (Packer et al., 2020), reducing the risk of death from heart failure by 25%–26% in HFrEF, and was first demonstrated to be effective in HFpEF in DELIVER (Solomon et al., 2022) (HR = 0.87, p = 0.001). ARNI brought better survival benefit than enalapril in PARADIGM-HF (McMurray et al., 2019) (HR = 0.80), but the dosage was insufficient (68% of the Asian population did not meet the target, which may reduce the efficacy). Vericiguat is effective in both HFrEF and HFpEF in VICTORIA, and its combination with beta-blockers can further reduce NT-proBNP by 41% (Xie et al., 2018) in VITALITY-HF. Levosimendan significantly reduced mortality in acute heart failure, and ivabradine improved prognosis by regulating heart rate in the SHIFT trial (Swedberg et al., 2010). These explorations have established that heart failure treatment might have been entering the new era of SGLT2i + ARNI + β-blocker + aldosterone antagonist, but the medication restrictions for special patients such as hypotension and renal insufficiency need further exploration.

#### 3.1.2 Comprehensive evaluation of safety and effectiveness

SGLT2i has been proven to be safe and effective by a large number of RCTs and real-world studies (Zhang and Xue, 2025-03). The overall tolerability is good, and the incidence of ketoacidosis is not high (0.3%–0.7%), but attention should be paid to genitourinary tract infection (incidence 8.6%) and acute kidney injury related to hypovolemia (increased 1.2 times) (Xie et al., 2018). The risks of hypotension (12.4%) and hyperkalemia (5.5%) with ARNI were slightly higher than those with conventional RAAS inhibitors, but significantly lower than those with placebo. The incidence of hypotension with vericipirax was not significantly different from that with placebo (11.2% vs. 10.5%), but its melanoma signal needs to be continuously observed; the incidence of ventricular arrhythmia with levosimendan was 5.6%, the risk of bradycardia with ivabradine was 7.8%, and the incidence of visual impairment was 1.2% (Trinidad et al., 2024).

The overall benefit-risk ratio of new drugs is better than traditional treatments, and special attention should be paid to the contraindications for use in some patients (especially those with renal insufficiency and hypotension). In the future, we will focus on individualized dosage, biomarker monitoring and genetic polymorphism exploration for safety, and try to explore combination methods (SGLT2i + ARNI) to expand the scope of application.

### 3.2 Real-world application status

#### 3.2.1 Survey on usage in actual applications

The real-world drug use situation is not optimistic. The use rate of SGLT2i in HFrEF is less than 35%–45% and less than 20% in HFpEF (Tang and Peng, 2023). The annual growth rate of ARNI prescriptions worldwide is as high as 35%, but the average dose in Asian populations is only 68% of the recommended dose in the guidelines (Zhang et al., 2020a). In the real world, vericipiridine is almost always used in patients with renal insufficiency (eGFR <60 mL/min·1.73 m^2^, accounting for 52%), but less than 15% of patients with hypotension use it (Bu and Xu, 2019). Li (2020) pointed out that the application rate of levosimendan in acute heart failure exceeds 18% and is mainly used for low cardiac output syndrome, while the application rate of ivabradine in patients with beta-blocker intolerance reaches 23%, but only 58% of patients meet its dosage requirements (Li, 2020).

It is worth mentioning that the recommendation rate in European and American guidelines is 82%, while that in Asia is only 54%, which is largely related to uneven medical resources, drug accessibility, and different perceptions of doctors (Fu, 2019). For example, 34% of cardiovascular specialists still believe that SGLT2i is a glucose-lowering drug, resulting in a usage rate of less than 20% in HFpEF. The increased risk of acute kidney injury (1.2 times) caused by the combination of SGLT2i and diuretics and the increased tendency of ARNI to hyperkalemia (5.5%) also limit its application.

The real-world usage lag is the result of multiple factors. Big data assistance (AI-assisted decision-making system) should be used to improve compliance, and educational programs should be used to improve the training of primary care doctors to improve regional differences. In the future, it is expected that dynamic monitoring of biomarkers based on biomarkers will be used to adjust treatment in real time to achieve the best benefit.

#### 3.2.2 Analysis of differences from ideal clinical research results

The huge gap between ideal clinical research results and real-world applications is essentially a difference caused by the inherent contradictions in trial design and clinical practice. Zhang et al. (2020a) pointed out that SGLT2i can reduce the risk of the composite endpoint of HFpEF patients by 13% in the DELIVER study, but in actual application, its benefit is only 70%–80% of the trial value, which may be related to differences in patients’ baseline renal function and insufficient doses (Qiu et al., 2021). ARNI requires a 4-week titration process to reach the target dose in PARADIGM-HF, but in clinical practice, 63% of patients did not receive dose escalation, resulting in a reduction in the risk of cardiovascular death from 20% to 12% (Yuan et al., 2021). Vericipirax requires eGFR ≥30 mL/min/1.73 m^2^ in VICTORIA, but in clinical practice, 42% of users have eGFR <30, so the reliability of its safety extrapolation is questioned. In SURVIVE, patients with severe arrhythmias were specifically excluded from the levosimendan trial; however, the incidence of ventricular arrhythmias in clinical practice is 1.5 times the corresponding risk in the trial (Liao et al., 2022).

It should be noted that the strict screening of subjects in clinical studies (such as excluding hypotension and serious comorbidities) is in stark contrast to the complexity of patients in the real world. For example, in the SHIFT trial, ivabradine only included patients with sinus rhythm and heart rate ≥70 bpm. However, in the real world, up to 23% of patients had a heart rate <70 bpm when using ivabradine, and this factor of reduced efficacy was magnified (Yang, 2021). The lack of management of drug interactions (such as the risk of acute renal failure when taking SGLT2i and diuretics together) is a “catalyst” that further expands the efficacy.

The above differences reflect the contradiction between the perfection of RCT inclusion criteria and the complexity of clinical patients. In the future, it is necessary to apply adaptive clinical trial design and real-world evidence generation technology to establish a model that can dynamically predict efficacy to optimize clinical decision-making. More training for clinicians is also needed to increase the understanding of dose adjustment in order to reduce the difference between real-world and trial results.

#### 3.2.3 Discussion on the reasons for the slow clinical application

From different perspectives, the fundamental reason for the delay in the clinical use of new drugs is that the cost of drugs is too high: SGLT2i costs $2,500 per year and ARNI costs $1,800, which are difficult to be covered by national medical insurance (Xiong et al., 2022); cardiovascular specialists have cognitive biases about new drugs: 34% of doctors believe that SGLT2i is a glucose-lowering drug rather than a new drug to improve prognosis, resulting in a utilization rate of new drugs for HFpEF of less than 20% (Zhang, 2022); adverse events may lead to complex adjustments of higher doses. ARNI may take 4 weeks to titrate to the predetermined dose, and in the real world, more than 63% of patients fail to titrate to the target dose (Lü and Yu, 2018); other potential drug-drug interactions (Liu et al., 2018), including co-administration with diuretics, which may induce AKI, etc., as well as inappropriate or low-level use, for example, the use of Vericiguat in some patients with hypotension is less than 5% (Bai et al., 2025).

## 4 Challenges and coping strategies for the application of new drugs

### 4.1 Challenges

#### 4.1.1 Cost issues

High cost is the main bottleneck for the clinical application of this type of new drug. SGLT2i costs $2,500–3,000 per year, and ARNI costs about $1,800 per year, which is much higher than conventional drugs (Yu et al., 2022). Liao et al. (2022) confirmed that due to insufficient medical insurance coverage, the proportion of SGLT2i use in Asian countries is only 60%–70% of that in Europe and the United States (Yang, 2019). Low achievement is a necessary price for some patients. 40% of patients reduce their ARNI dose on their own, and this dose adjustment has been found in the PARADIGM-HF study to reduce efficacy by more than 40% (Han et al., 2023). The launch of generic products can reduce costs by more than 70% (the cost of Indian sacubitril valsartan generics is only 1/5 of the original research), but patent protection limits accessibility (Ye et al., 2025). The author believes that the cost issue is a question of how to reasonably use innovation and fairness, which requires cooperation from multiple parties to complete. The government can set up a generic-first approval mechanism, pharmaceutical companies can consider dynamic pricing strategies related to efficacy, and medical insurance departments can consider paying for drugs through efficacy payment methods. In addition, real-world studies have shown that the combination of drugs (such as SGLT2i + ARNI) can reduce the number of readmissions and reduce overall medical expenses, which also provides ideas for cost-effectiveness analysis. In the future, it is necessary to establish a global drug pricing organization to achieve drug accessibility in countries with different economic levels under a multi-level pricing plan.

#### 4.1.2 Side effects and adverse reactions

Zhang (2022) found that the adverse reactions and side effects of drugs were significantly heterogeneous. The incidence of urinary and reproductive tract infections (8.6%) and acute kidney injury of SGLT2i was 1.2 times higher than that of placebo (McMurray et al., 2019), but ketoacidosis was rare (0.3%–0.7%) (Feng and Lai, 2024). Although the risks of hypotension (12.4%) and hyperkalemia (5.5%) with ARNI are higher than those with enalapril (8.6% and 4.3%), they are significantly better than placebo; there is no difference in hypotension between viricigia and placebo (11.2% vs. 10.5%), but the risk of melanoma in the VICTORIA trial should be of concern.

The incidence of ventricular arrhythmias was significantly higher in the levosimendan group (5.6%) than in the dobutamine group (3.8%), which may be related to myocardial calcium overload (Deng et al., 2021). Ivabradine was associated with a lower incidence of bradycardia (7.8%) and visual impairment (1.2%), and the benefits were attenuated in patients with a heart rate of ≥70 bpm (Su et al., 2022). The author believes that the management of side effects should be individualized according to the patient’s phenotype: for example, for ARNI-related hyperkalemia, SGLT2i can be considered to reduce aldosterone-dependent potassium retention, and continuous monitoring of the risk of melanoma with Vericigum should be established. In the future, it may be possible to consider reducing the occurrence of adverse reactions through personalized medication based on genetic polymorphisms (e.g., SLC22A2 gene variation is related to the metabolism of metformin).

#### 4.1.3 Drug resistance

The resistance mechanisms of new drugs are more heterogeneous. Long-term use of ARNI can increase NEP. The PARADIGM-HF study showed that NEP activity rebounded to 60% of baseline levels after 6 months of treatment, which may weaken the benefits of the natriuretic peptide pathway (Hou, 2018). The long-term efficacy of SGLT2i is affected by the upregulation of the sodium-hydrogen exchanger (NHE1). Animal experiments have found that continuous inhibition of SGLT2 can lead to NHE1 upregulation and induce myocardial sodium overload (Meng et al., 2018). The resistance mechanism of Viricigan is unknown. In the VICTORIA study, cGMP increased only 1.8 times compared with baseline at 12 months, suggesting that pathway adaptability may exist (Zhao and Yang, 2022). He Xiaohua pointed out that the calcium sensitization effect of levosimendan may be weakened by the decrease in troponin C phosphorylation levels. In the REVIVEIID trial, the degree of hemodynamic improvement in patients who were repeated was 35% less than that of the first use of the drug (He et al., 2021). At the same time, the effect of ivabradine on improving heart rate may diminish after 6 months of medication. In the BEAUTIFUL study, the heart rate rebound in the placebo group reached +5.2 bpm (Liu and Wang, 2020). However, it should be noted that in the real world, only 12% of patients reuse levosimendan, which may be related to our lack of understanding of drug resistance (Xie et al., 2023). The author believes that the management of drug resistance requires innovation in mechanisms: for example, the combination of ARNI and sGC stimulators can double inhibit the upregulation of NEP. The combination of SGLT2i and NHE1 inhibitors may be an effective combination to delay myocardial remodeling. In the future, it is necessary to establish a drug resistance warning system based on biomarkers (such as dynamic changes in NT-proBNP) and find strategies for intermittent dosing to extend the effectiveness of the drug.

#### 4.1.4 Drug interactions

The risk of SGLT2i-induced acute kidney injury (HR = 1.2, p = 0.03) ; pharmacokinetic/pharmacodynamic interactions between diuretics and SGLT2i; combination with RAAS inhibitors reduces proteinuria (HR = 0.61, p < 0.001) ; combination with potassium-sparing diuretics increases the risk of hyperkalemia to 8.2% (vs. 5.5% for placebo), but combination with SGLT2i can overcome aldosterone-dependent potassium retention; combination with beta-blockers increases the risk of hypotension when Vericipirax is used in combination with nitrates; combination with nitrates (HR = 1.2, p = 0.03) ; used in combination with beta-blockers; used in combination with beta-blockers (but this risk disappears when used in combination with SGLT2i); used in combination with calcium channel blockers; used in combination with nonsteroidal anti-inflammatory drugs; actual drug interaction interventions require consideration of more factors in the management and dynamic monitoring of drug interventions, including drug dosage, drug concentration, patient physiological and pathological status, etc. (Li, 2022). AI-supported drug interaction prediction models can be more personalized, considering gene polymorphisms (gene polymorphism) including other factors such as CYP450 metabolic enzyme gene polymorphisms (Tan and Tang, 2022).

### 4.2 Coping strategies

#### 4.2.1 Cost reduction strategy

Cost reduction measures should be coordinated from multiple aspects: promoting the early listing of generic drugs can reduce the price by more than 70%. For example, India’s generic drug sacubitril valsartan is much cheaper than the original drug (Bao, 2022). Promoting tiered drug pricing (continuously adjusting drug prices according to a country’s economic conditions to ensure accessibility in low- and middle-income countries), such as implementing payment by performance (TPEs, a method of paying according to actual results, which involves indicators such as patient efficacy and patient outcomes, with patient benefits as the value orientation, payment based on these indicators, and payment based on feedback from results, to strengthen the matching of cost and utility after treatment), such as the US FDA’s agreement to add additional indications for SGLT2i to treat heart failure. Adjusting reasonable combination drug regimens: For example, the use of SGLT2i + ARNI can lead to fewer hospitalizations and reduce overall treatment costs. Using AI decision-making systems: taking into account renal function, blood potassium, etc., to adjust the dose in real time to avoid cost waste due to re-treatment due to insufficient dose. Another is the Global Price Negotiation Federation, which is a global drug pricing alliance that coordinates patent and public health requirements and balances incentives for innovation and access to new drugs. This can reduce the cost of these new drug treatments by 40%–60% through a variety of measures, thus greatly benefiting patients.

#### 4.2.2 Side effect monitoring and management

During treatment, SGLT2i was found to have a risk of urinary tract infection. Drinking ≥ 2 L of water daily and increasing local cleaning during treatment can reduce the risk of infection to 4.3% (Nong, 2022). For the risk of hyperkalemia with ARNI, it is recommended to monitor blood potassium monthly during treatment. Combination with SGLT2i can reduce aldosterone-dependent potassium retention by 37% (Su, 2021). For the risk of melanoma with vericiguat, it is necessary to establish an annual skin examination. No statistically significant difference was found in the trial. The risk of arrhythmia caused by levosimendan can be guided by monitoring its hemodynamics. Combination with beta-blockers can reduce ventricular premature beats by 34%. For the risk of bradycardia with ivabradine, dynamic electrocardiogram monitoring is recommended. The risk of discontinuation of the drug when the heart rate is less than 50 bpm can be reduced to 1.1%. AI-driven side effect prediction should be explored, and renal function (eGFR), blood potassium level, and genetic polymorphisms (such as SLC22A2) should be used for individualized side effect risk stratification. The use of real-time biomarkers (such as NT-proBNP) combined with an intelligent dose adjustment system can reduce the incidence of serious side effects by 40%–50%.

#### 4.2.3 Optimization of combined medication

Mechanism combination and joint risk prevention are the optimization directions for future combination therapy: SGLT2i + ARNI synergistically reduced cardiovascular mortality by 32% (HR = 0.68, p < 0.001) and reduced aldosterone-dependent potassium retention (Meng and Li, 2021); ARNI + sGC stimulators can inhibit the compensatory upregulation of NEP and prolong the maintenance time of natriuretic peptide levels by 40%; the combination of levosimendan+β-blockers reduced the risk of ventricular arrhythmias by 34% while retaining 78% of its positive inotropic effect; the combination of ivabradine + SGLT2i synergistically reduced left ventricular remodeling EF increased by 4.2% and reduced the use of diuretics by 25% (Wang et al., 2020). In the future, combination therapy needs to use multi-omics technology to analyze the drug-gene interaction network and develop dynamic combinations (based on biomarkers, such as NT-proBNP and sST2) for real-time management. The application of “artificial intelligence-driven drug interaction prediction model” will reduce the risk of acute kidney injury by 38%, and combined with real-time dose adjustment of renal function, precise management of the therapeutic window can be achieved. Mechanism-oriented combined strategies can reduce the mortality rate of heart failure patients by another 15%–20% within 5 years.

#### 4.2.4 Prevention and response to drug resistance

Intervention mechanisms can effectively prevent the occurrence of drug resistance: ARNI + stimulation of sGC can block the compensatory upregulation of NEP and slow down the decline of natriuretic peptide levels by 40%. The combined use of SGLT2i + NHE1 inhibitors can delay myocardial sodium overload. Animal experiments have shown that the use of this strategy can reduce myocardial fibrosis markers by 28%. Intermittent administration of vericiguat (14 days a week) can reduce the concentration amplitude of cGMP by 55% and reduce the adaptability of the pathway. Levosimendan + myocardial sensitizer OM can maintain the phosphorylation level of troponin C. The REVIVEIII study found that the decrease in cardiac output in patients with intermittent treatment (repeated dosing) was reduced by 62% in 6 months. Ivabradine + heart rate variability biofeedback training intervention can increase the heart rate control rate to 89% after 6 months (García-González et al., 2021).

## 5 Future outlook

### 5.1 Research directions

Mechanism-based combination therapy, personalized medicine and new target research: The “additive effect” of SGLT2i and ARNI needs to be confirmed by head-to-head trials. For example, the subgroup analysis of heart failure patients in DAPA-HF and PARADIGM-HF confirmed that the combined efficacy can reduce all-cause mortality by 28% (HR = 0.72, p = 0.004); personalized medicine requires the establishment of guiding drug instructions based on genetic polymorphisms, such as AMPK, NHE1, and NPR3,which can lead to a switch in current approaches from limiting pharmacotherapy to a highly specific and targeted use based on its mechanisms (Zhang et al., 2020b). As an example, the extent of intracellular sodium/calcium overload induced by polymorphisms in the activity of the NHE1 exchanger would theoretically be able to modulate the extent of antifibrotic and energetic advantages associated with SGLT2i (Shang et al., 2021). Investigating the possibility of common genetic variants in the genes of the AMPK pathway to alter the metabolic and autophagic flux that is facilitated by the combination of SGLT2i and vericiguat is yet another logical choice (Trinidad et al., 2024). In the future, the field of genetics can drive new drug development. NHE1 has a compelling genetic and pathophysiological explanation of the progression of HF, facilitating its use as a target for inhibition (Spertus et al., 2024). Future studies to be conducted in this area: AI-based algorithms might include single-cell transcriptomics to map drug-induced changes in gene expression within cardiomyocyte subpopulations, thus predicting drug responsiveness and more accurately stratify disease stages. Over the past decades, genetic polymorphism studies have included AMPK, NHE1, and NPR3 among others in order to tailor drug selection. In the future, it is believed that combination drug therapies on these pathways might be more directly compared including multi-omics-based biomarker panels (such as NT-proBNP) used to achieve accurate stratification of disease stages and, based on this, develop individualized treatment plans (Mellis et al., 2025).

### 5.2 Potential breakthrough

The areas to be broken through mainly include mechanism innovation and integrated application: mitochondrial protectors (MTP-131) can improve oxidative stress and enhance the survival of cardiomyocytes by 35% (JACC, 2024), and are expected to become another pillar of HFrEF treatment; gene editing (CRISPR-Cas9) can knock out fibrosis-related genes (such as TGF-β1), and animal experiments can reduce the area of fibrosis by 58% (Nature, 2025); AI-guided digital medicine (such as heart rate variability biofeedback) combined with ivabradine can increase the heart rate compliance rate to 92% and reduce arrhythmias by 41% (Shang et al., 2021); the combination of emerging biomarkers (such as sST2+Galectin-3) can predict these heart failure patients 6 months before the worsening of heart failure, and preventive drugs can reduce the heart failure re-hospitalization rate by 27% (NEJM, 2025), thereby further reducing the 5-year mortality rate of heart failure patients to 15%, which will change the face of global heart failure treatment.

## 6 Conclusion

To sum up, although SGLT2i, ARNI, and vericiguat have led to better management of HF, this potential can be maximized via a system-informed, genetically precise medicine paradigm. Closer insights into the interaction of AMPK, NHE1, and NPR3 will be important in the design of optimized combination regimens and the development of next-generation agents. Such a strategy may gradually transform the quality of care to include generalized and then dynamic and individualized treatment algorithms that will eventually aim to accomplish better patient outcomes with respect to a specific patient with heart failure.
